# Albumins represent highly cross-reactive animal allergens

**DOI:** 10.3389/fimmu.2023.1241518

**Published:** 2023-10-20

**Authors:** Zicheng Liu, Daria Trifonova, Inna Tulaeva, Ksenja Riabova, Antonina Karsonova, Evgeny Kozlov, Olga Elisyutina, Musa Khaitov, Margarete Focke-Tejkl, Ting-Huan Chen, Alexander Karaulov, Rudolf Valenta

**Affiliations:** ^1^ Division of Immunopathology, Department of Pathophysiology and Allergy Research, Center for Pathophysiology, Infectiology and Immunology, Medical University of Vienna, Vienna, Austria; ^2^ Laboratory for Immunopathology, Department of Clinical Immunology and Allergology, Sechenov First Moscow State Medical University, Moscow, Russia; ^3^ National Research Center, NRCI Institute of Immunology, Federal Medical-Biological Agency (FMBA) of Russia, Moscow, Russia; ^4^ RUDN University, Moscow, Russia; ^5^ Pirogov Russian National Research Medical University, Moscow, Russia; ^6^ Karl Landsteiner University of Healthcare, Krems, Austria; ^7^ Worg Pharmaceuticals, Hangzhou, China

**Keywords:** allergy, allergen, epitope, albumin, cross-reactivity, tolerance, antibody, immunoglobulin E (IgE)

## Abstract

Albumins from animals are highly cross-reactive allergens for patients suffering from immunoglobulin E (IgE)-mediated allergy. Approximately 20-30% of cat and dog allergic patients show IgE reactivity and mount IgE-mediated allergic reactions to cat and dog albumin. It is astonishing that allergic patients can develop specific IgE responses against animal albumins because these proteins exhibit a more than 70% sequence identity to human serum albumin (HSA) which is the most abundant protein in the blood of the human body. The sequence identity of cat albumin (Fel d 2) and dog albumin (Can f 3) and HSA are 82% and 80%, respectively. Given the high degree of sequence identity between the latter two allergens and HSA one would expect that immunological tolerance would prohibit IgE sensitization to Fel d 2 and Can f 3. Here we discuss two possibilities for how IgE sensitization to Fel d 2 and Can f 3 may develop. One possibility is the failed development of immune tolerance in albumin-allergic patients whereas the other possibility is highly selective immune tolerance to HSA but not to Fel d 2 and Can f 3. If the first assumption is correct it should be possible to detect HSA-specific T cell responses and HSA-containing immune complexes in sensitized patients. In the latter scenario few differences in the sequences of Fel d 2 and Can f 3 as compared to HSA would be responsible for the development of selective T cell and B cell responses towards Fel d 2 as well as Can f 3. However, the immunological mechanisms of albumin sensitization have not yet been investigated in detail although this will be important for the development of allergen-specific prevention and allergen-specific immunotherapy (AIT) strategies for allergy to albumin.

## Introduction

IgE-mediated allergy to animals, in particular to cats is very common (1). A recent survey of the molecular IgE sensitization profiles in sera collected from more than 2800 children in the course of longitudinal birth cohort studies in different European regions identified the major cat allergen Fel d 1 as one of the most frequently recognized allergens (2). While Fel d 1 is an allergen that is highly specific for cats (3, 4), animals also contain cross-reactive allergens among which the serum protein albumin is the most important cross-reactive allergen known to date (5). Albumin had been described as an allergen in dogs and cats as long as 50 years ago (6, 7). In the years since, recombinant albumin allergens from dogs and cats were obtained by molecular cloning techniques. Using serum IgE from dog-allergic patients, a cDNA coding for a recombinant dog albumin fragment was isolated from a cDNA library constructed from dog liver mRNA (8). Recombinant dog albumin expressed in *Escherichia coli* (*E. coli*) has then been shown to cross-react with albumins from several different animals (9). Additionally, cat albumin could be expressed as a recombinant allergen in *E. coli*, and IgE recognition of Fel d 2 was found to be frequent in cat-allergic patients suffering from atopic dermatitis (10). The relevance of albumin as an allergen was demonstrated by its ability to induce specific basophil activation and immunization of mice with albumin-induced strong specific IgE sensitization (10, 11). In population-based birth cohort studies cat and dog albumin have been identified as frequently recognized allergens (12). The Swedish BAMSE birth cohort study showed a progressive increase in the prevalence of IgE sensitization to Fel d 2 and Can f 3. IgE sensitization to Fel d 2 was much more common than to Can f 3 and was detected in approximately 7% of all of the investigated children at the age of 16 years (12).

IgE cross-reactivity towards albumins from different animals has been demonstrated (9) and IgE co-sensitization has been investigated using micro-arrayed albumins from different animals by IgE serology (13). Although albumin is not the immune-dominant allergen in most of the patients suffering from animal allergy it accounts for the majority of animal-specific IgE in certain patients (13). Interestingly, T cell reactivity to albumin allergens in allergic patients has so far not been investigated in detail.

## Albumins are highly abundant serum proteins with conserved primary and tertiary structure in animals and humans

Albumin belongs to a family of globular transport proteins (i.e., serum albumin, alpha-fetoprotein, Vitamin D-binding protein, afamin, extracellular matrix protein 1) in the blood which can bind various endogenous, exogenous ligands as well as drugs (14, 15) (https://albumin.org/, https://en.wikipedia.org/wiki/Albumin). Albumins are water soluble and in contrast to many other blood proteins are not glycosylated. The word albumin has its origins in the word “albumen”, which means protein, and the Latin root word “albus” meaning white, and refers to the white color of egg white when eggs are cooked. Serum albumin is synthesized in and secreted from liver cells as a single-chain protein. Human serum albumin consists of 585 amino acids and according to its sequence has a molecular mass of 66,500 Da (16). Human serum albumin contains a large number of acidic and basic amino acid residues and hence is highly soluble in aqueous solutions. As long ago as the 1930s, T. Svedberg and K. O. Petersen in Uppsala determined its molecular weight to be 66.5 kDa by ultracentrifugation (17). As the most abundant storage and transport protein in the body’s circulatory system (18), albumin has many important physiological and pharmacological functions and can be combined with many endogenous and exogenous substances such as fatty acids, amino acids, hormones, anions, cations, and drugs (19). The physiological functions of albumin include transporting ligands, maintaining plasma osmotic pressure, anti-oxidative and free radical scavenging and it is also used in disease treatment and diagnosis (20).

The three-dimensional structure of HSA as revealed by x-ray crystallography shows that the protein contains three structurally similar functional domains (domain I, II, III) which assemble in the form of a heart-shaped protein (21). Each functional domain (I-III) contains two subdomains (subdomain A and subdomain B). The hydrophobic cavities in subdomains IIA and IIIA are mainly responsible for ligand binding. The structure of HSA is stabilized by 17 disulfide bridges distributed in each tertiary region, as well as one free thiol group (21). This architecture is similar among serum albumins, in which each domain contains five or six internal disulfide bonds.

As already indicated, serum albumin plays an important role in the pharmacokinetics of drugs, especially their distribution in the human body. In 1975, G. Sudlow discovered two different types of sites on serum albumin where dansyl-L-asparagine (DNSA) and dansylsarcosirie could be displaced by drugs using fluorescent probe displacement assays (22). Fatty acids, warfarin, antioxidant metal ions, nitric oxide, and oxygen are possible ligands for the serum albumin binding sites (23–26). Most of the drugs first combine with serum albumin, and then reach the target tissue for storage and further transportation. Since albumin is synthesized in the liver, liver damage, malnutrition, and inflammation may lead to alterations in the rate of albumin synthesis (20).

## Albumins as cross-reactive allergens but cross-reactivity can be limited: importance for allergy diagnosis

**Figure 1:**
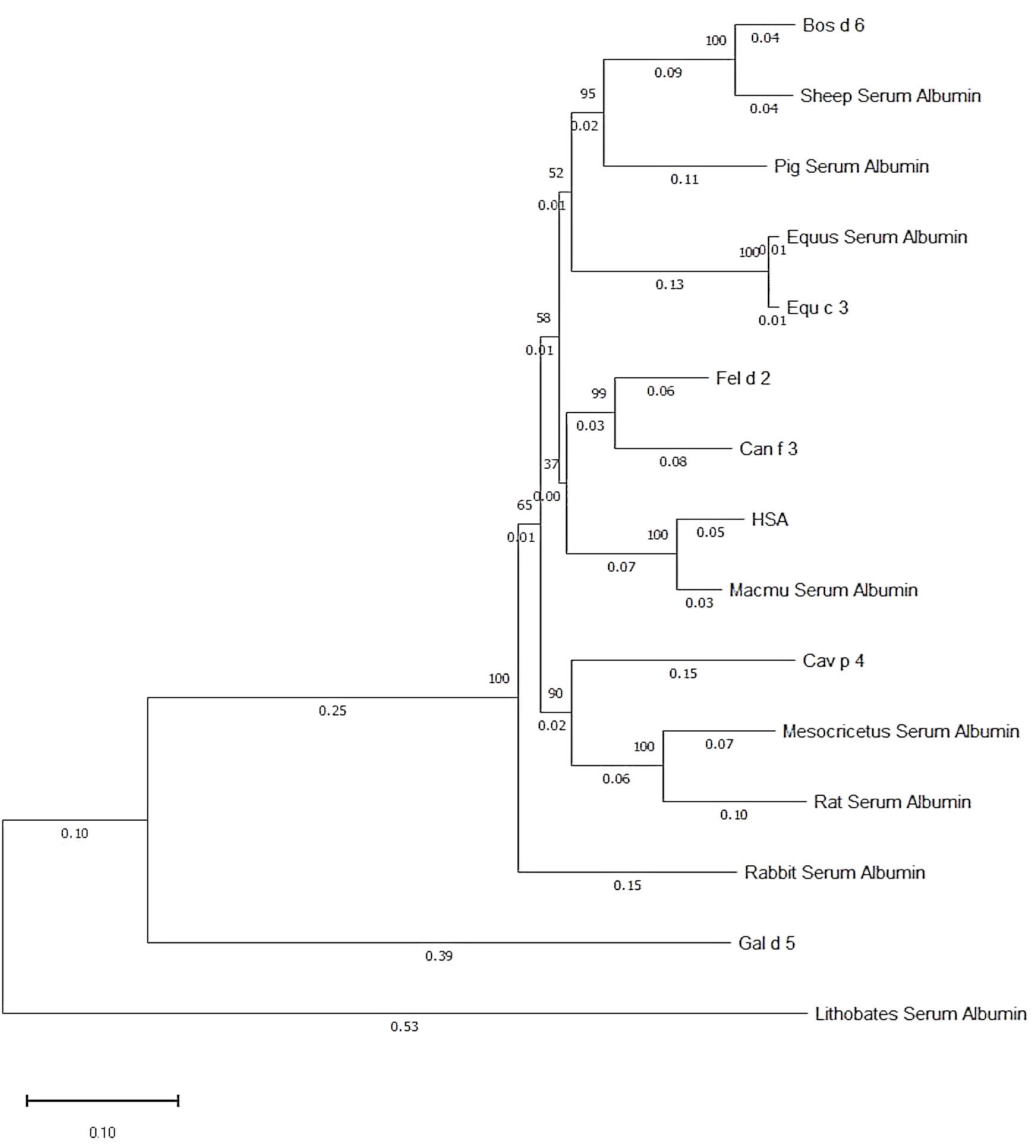
Phylogenetic tree of albumins from different sources. The numbers on the branch point indicate phylogenetic likelihood, whereas the numbers on the branch line indicate phylogenetic distance to branch point. Generated by Molecular Evolutionary Genetics Analysis software.

**Figure 2:**
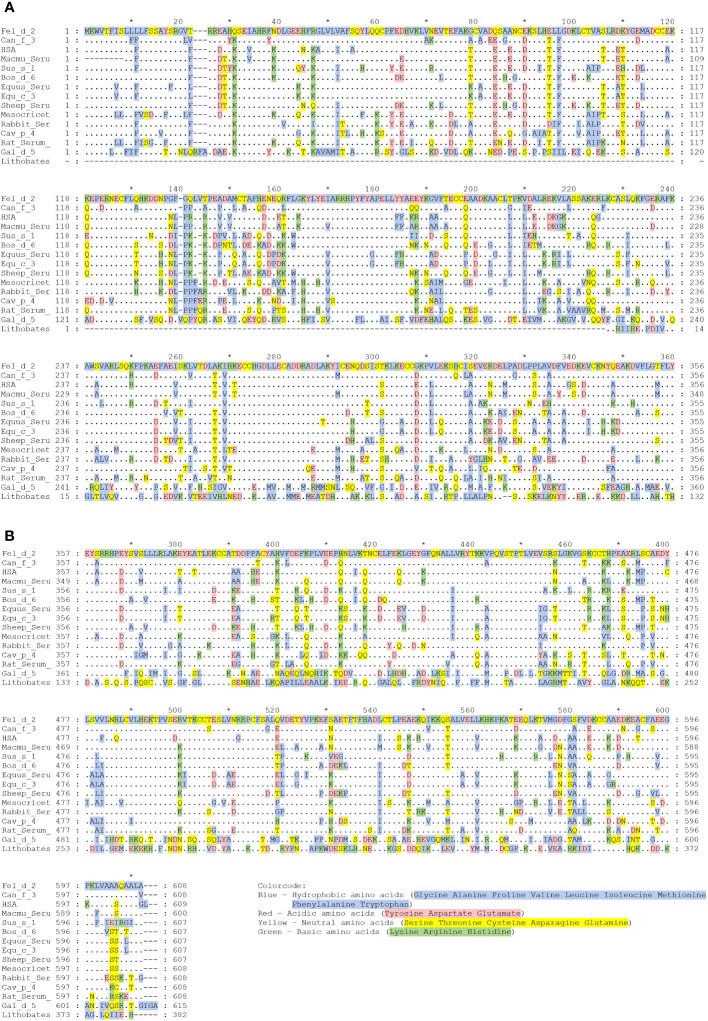
**(A, B)** Alignment of the amino acid sequence of HSA with albumins from other sources. Amino acid numbers are shown on the right and left margin and designations of albumins are given at the right margin, which are from top to bottom: Human, rhesus macaque, cat, dog, donkey, horse, pig, bovine, golden hamster, sheep, rabbit, rat, guinea pig, chicken, and American bullfrog. Amino acids identical to Fel d 2 are shown as dots, dashes indicate gaps. Amino acids are colored according to their chemical properties: Blue – Hydrophobic amino acids (Glycine, Alanine, Proline, Valine, Leucine, Isoleucine, Methionine, Phenylalanine, Tryptophan); Red – Acidic amino acids (Tyrosine, Aspartic acid, Glutamic acid); Yellow – Neutral amino acids (Serine, Threonine, Cysteine, Asparagine, Glutamine); Green – Basic amino acids (Lysine, Arginine, Histidine).

Fel d 2 (i.e., cat albumin) is the most frequently recognized albumin allergen (2, 27). It consists of 608 amino acids including a signal peptide comprising the first 18 amino acids, 29 alpha helices, 4 turns, and 2 beta strands (see IUIS Allergen nomenclature database: http://www.allergen.org/viewallergen.php?aid=320; Protein sequence: https://www.ncbi.nlm.nih.gov/protein/CAA59279). If one constructs a phylogenetic tree displaying evolutionary relationships of albumins from various species based on sequence features (**Figure 1**) it can be noticed that Fel d 2 is represented on a branch together with dog albumin, Can f 2, and HSA (**Figure 1**). The relationship of albumins displayed in the phylogenetic tree (**Figure 1**) and sequence similarity can be also observed in the alignment of amino acid sequences (**Figures 2A, B**). When sequences are aligned according to similarity with HSA on top, the alignment reflects to a large extent the previously observed cross-reactivity of IgE antibodies investigated in earlier immunological studies (8). These studies showed a high degree of IgE cross-reactivity between cat and dog albumin whereas IgE cross-reactivity between cat and dog albumin and bovine serum albumin, horse albumin, rat albumin, and chicken albumin (Gal d 5) were lower, respectively. However, so far no relevant IgE reactivity to HSA could be demonstrated in patients sensitized to cat and dog albumin, although Fel d 2, Can f 3 and HSA showed a high degree of sequence identity and are phylogenetically closely related (**Figures 1**, **2A, B**). This may be due to the fact that human serum contains large amounts of HSA which will block HSA-specific IgE from binding to solid-phase bound HSA. In fact, at the level of primary structure, cat, dog, and human serum albumin all have 35 cysteines forming 17 pairs of disulfide bonds, similar to the C-(X)18-C-(X)8-C-(X)12-C-(X)14-CC-(X)9-C-(X)22-C-(X)43-CC-(X)30-C-(X)44 pattern. This highly conserved primary structure gives albumin the ability to form alpha helices, ensuring the stability of the protein. Thereby, in the tertiary structure, albumin presents an extremely stable globulin structure composed entirely of alpha helices. In the circulatory system, when the pH and other physicochemical parameters change, the large number of disulfide bonds can help albumin to reversibly change its structure, thus enabling numerous physiological functions (19). The amino acid sequences of albumin are 70% or more identical between mammals whereas sequence identities with chicken albumin are below 50% (**Table 1**). Again the comparison of sequence identities shows that HSA has a higher overall sequence identity to Fel d 2 and Can f 3 as compared to allergenic albumins from other albumins (**Table 1**). Nevertheless, IgE reactivity to albumins shown in **Table 2** has been reported whereas no relevant IgE reactivity to HSA has been found in allergic patients (6, 28–44). Thus the question arises if in addition to overall sequence identity few high-impact amino acid exchanges that may affect sequential and/or conformational epitopes may have dramatic effects on IgE cross-reactivity to albumins. This would eventually explain an exquisite lack of IgE reactivity to HSA.

**Table 1:** Sequence identities of albumins from different sources.

Albumin/Allergen name	Fel d 2														
Fel d 2	100	Can f 3													
Can f 3	87	100	HSA												
HSA	82	80	100	Macmu											
Macmu Albumin	81	81	93	100	Sus s 1										
Sus s 1	79	78	76	76	100	Bos d 6									
Bos d 6	79	77	76	77	80	100	Donkey								
Donkey Albumin	78	76	77	78	77	74	100	Equ c 3							
Equ c 3	78	76	76	78	76	75	99	100	Sheep						
Sheep Albumin	78	78	75	76	78	92	76	76	100	Hamster					
Hamster Albumin	76	74	76	78	73	70	75	75	70	100	Rabbit				
Rabbit Albumin	76	75	75	76	75	72	71	72	73	74	100	Cav p 4			
Cav p 4	76	74	73	74	73	71	73	72	71	75	73	100	Rat		
Rat Albumin	75	73	73	74	72	70	72	72	70	84	73	74	100	Gal d 5	
Gal d 5	47	48	47	47	44	45	44	44	45	44	46	45	45	100	Lithobates
Lithobates Albumin	36	36	36	38	37	35	35	35	36	35	36	34	34	36	100

Sequence identities of albumins from different sources. Albumins have been ordered from top to bottom according to the highest sequence identity to Fel d 2 (top). Grey color indicates 100% sequence identity followed by red to yellow with declining identity. Percentage sequence identities among the albumins have been calculated by CLUSTAL alignment.

**Table 2:** Albumin allergens registered in the IUIS database and/or described in published studies.

Allergen source	Allergen name	Isoallergen and variants	GenBank Nucleotide ID	GenBank Protein ID	UniProt ID	PDB ID	Relevant references
*Cat*	Fel d 2	Fel d 2.0101	X84842	CAA59279	P49064	5YXE	(28)
*Dog*	Can f 3	Can f 3.0101	AB090854	BAC10663	P49822	5GHK	(6, 29, 30)
*Pig*	Sus s 1	Sus s 1.0101	M36787	AAA30988.1	P08835	\	(28, 31)
*Bovine*	Bos d 6	Bos d 6.0101	M73993	AAA51411	P02769	4F5S	(32–34)
*Buffalo*	\	\	\	\	\	\	(35)
*Donkey*	\	\	AY754333	AAV28861.1	Q5XLE4	\	(35)
*Horse*	Equ c 3	Equ c 3.0101	X74045	CAA52194	P35747	1UOR	(36, 37)
*Sheep*	\	\	X17055	CAA34903.1	P14639	6HN0	(35)
*Goat*	\	\	\	\	A0A452F7K4	6HN1	(35)
*Hamster*	\	\	EF488484	ABR68005.1	A6YF56	\	(38)
*Rabbit*	Ory c 1	\	U18344	AAB58347.2	P49065	6OCK	
*Guinea pig*	Cav p 4	Cav p 4.0101	AY294645	AAQ20088	Q6WDN9	\	(39–41)
*Rat*	\	\	V01222	CAA24532.1	P02770	\	(42)
*Mouse*	\	\	AK131891	BAE20854.1	Q546G4	\	(40, 43)
*Chicken*	Gal d 5	Gal d 5.0101	X60688	CAA43098	P19121	\	(44)

In this context, the example of pathogenesis-related allergens present in birch pollen (e.g., Bet v 1) and other plants, also known as PR10 allergens comes into mind (45, 46). IgE cross-reactivity between Bet v 1, related pollen allergens from trees belonging to the order *Fagales* (e., birch, alder, hazel, hornbeam), and PR10 allergens expressed in somatic plant tissues (e.g., fruits, seeds, and leaves) has been extensively described and explains varying degrees of allergic symptoms in PR10-sensitized patients (47, 48). As for albumins, IgE cross-reactivity to PR10 allergens follows more or less the overall sequence identities of the proteins, the more related to Bet v 1, the greater is the extent of IgE cross-reactivity because for most patients birch pollen and the therein contained Bet v 1 allergen, represents the genuinely sensitizing allergen source and allergen, respectively (49).

Likewise, differences in IgE reactivity in terms of specific IgE levels and presumably affinities/avidities of IgE antibodies to albumins have been observed (13). Accordingly, it has been suggested that differences in IgE reactivity to albumins from different sources may be useful for the identification of the genuinely sensitizing allergen sources among animals inducing respiratory sensitization (e.g., cats, dogs, horses) (13). Differences in IgE reactivity seem to play an even more important role when it comes to the question of whether a patient is sensitized primarily to a respiratory allergen source (e.g., cat, dog) and shows much lower secondary IgE reactivity to the corresponding allergen present in food (e.g., cow´s milk albumin) (50, 51). In this context, it has been shown, that based on allergen extract-based tests one may by mistake consider patients with IgE reactivity to BSA-containing milk as being cow´s-milk allergic although these patients were genuinely sensitized by respiratory sensitization to cat and/or dog dander (50). On the other hand, it has been demonstrated that strong IgE reactivity to BSA and BSA fragments may be indicative of primary sensitization to cow´s milk, especially in children (51).

**Figure 3:**
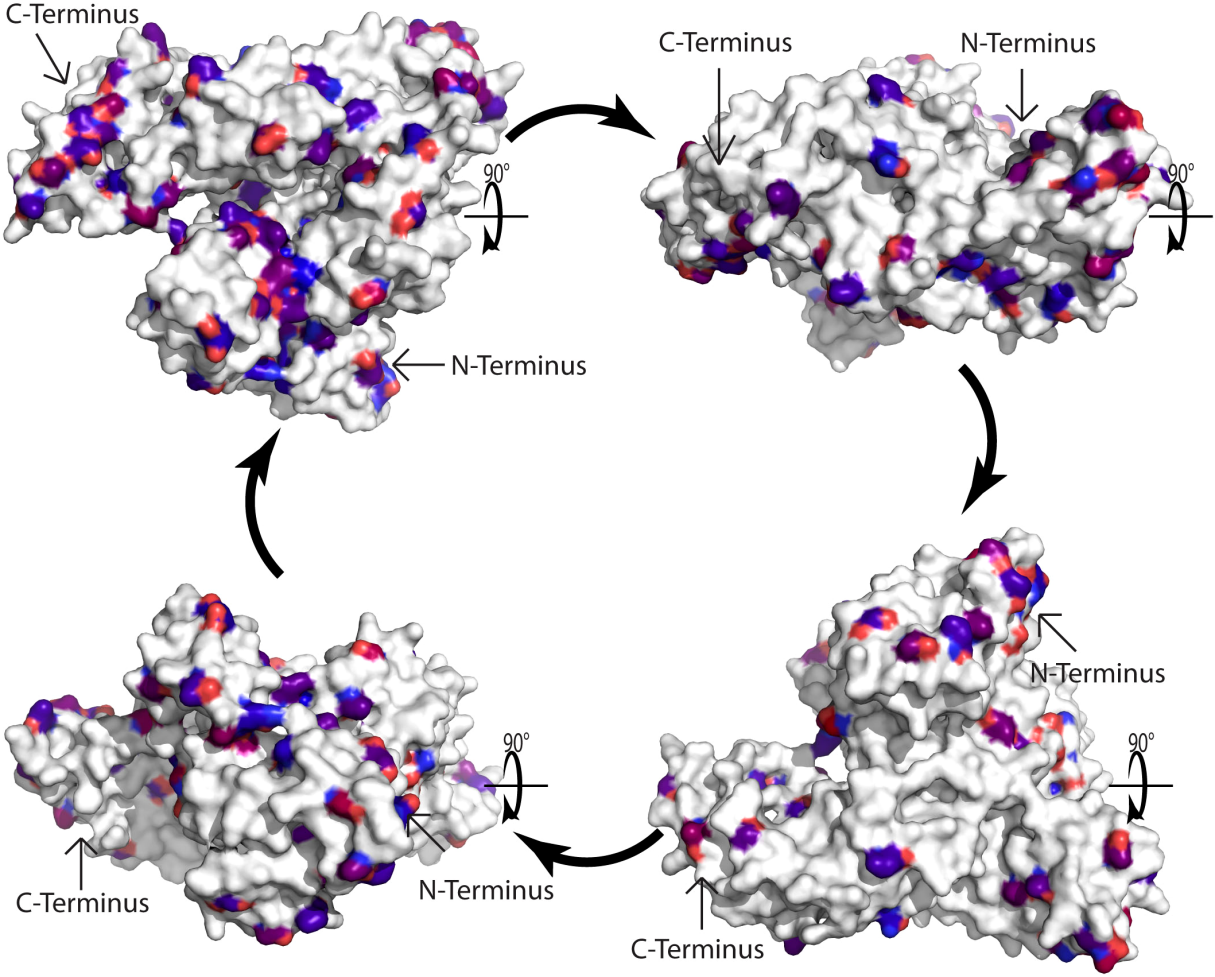
Model of the three-dimensional cat albumin structure. Surface representation of the model of the 3D structure of cat albumin (5yxe) as compared to human serum albumin (1ao6). In total 584 amino acids in Fel d 2 and 588 amino acids in Human serum albumin were aligned. Atoms with RMSD less than 0.972 Å were excluded. White amino acids indicate conservative regions identical to human serum albumin. Differences are represented by red and blue colors, with blue indicating more conserved mutations and red indicating less conserved mutations according to the BLOSUM90 substitution matrix. Arrows indicate the C- and N-terminus.

The remaining question is why there is no relevant IgE reactivity to HSA in Fel d 2 and Can f 3-sensitized patients. Again, one possible answer to this may come from previous observations made for the cross-reactive family of the Bet v 1-related PR10 allergen family. In fact, when Cor a 1, the major allergen of hazel pollen, was characterized at the molecular level by PCR and cDNA cloning it was realized that hazel pollen contains highly IgE-reactive and allergenic Cor a 1 isoforms but at the same time, basically non-IgE reactive Cor a 1 isoforms were discovered which differed from the highly allergenic Cor a 1 isoforms only by few important amino acid exchanges (52). Likewise, highly reactive Bet v 1 isoforms were discovered which also differed only by relatively few amino acid exchanges from hypoallergenic Bet v 1 isoforms (53–55). It is therefore tempting to speculate, that one possible explanation for the lack of relevant IgE reactivity to HSA could be the occurrence of relatively few but critical differences regarding amino acids between HSA and Fel d 2 or Can f 3. In the alignment of the amino acid sequences of albumin allergens and HSA in **Figure 2** we show by marking in different colors that such amino acid differences which may affect the surface charge or conformation (e.g., acidic versus basic, hydrophilic versus hydrophobic, non-conserved cysteine residues) indeed exist in Fel d 2, Can f 3 and HSA. Subsequently, we visualized amino acid differences in the model of the three-dimensional structures of Fel d 2 and HSA (**Figure 3**). Calculation of the hydrophilicity of the primary structure of Fel d 2 compared with HSA according to the Kyte & Doolittle algorithm showed that most (64 of 79) relevant amino acid exchanges of Fel d 2 versus HSA appeared to be in the hydrophilic fraction and accordingly can be visualized in the model of the three-dimensional structure of Fel d 2 (**Figure 3**). However, it has not yet been investigated if these amino acid exchanges indeed contribute to the observed differences in IgE reactivity. One possibility to investigate the effect of amino acid differences between Fel d 2 and HSA on IgE reactivity would be to conduct an IgE epitope mapping of Fel d 2 and subsequently mutate amino acids critical for IgE reactivity in Fel d 2 in HSA in order to try to render HSA IgE-reactive.

## IgE sensitization to albumin may occur via different routes and can exhibit different clinical phenotypes of allergy

**Table 2** provides an overview of albumins from different sources which have been shown to be recognized by IgE antibodies from allergic patients together with their primary and if available three-dimensional structure data and corresponding references (6, 28–44). According to population-based molecular IgE reactivity profiling available thus far, Fel d 2 and Can f 3 belong to the most frequently IgE-reactive albumins (2, 12). It thus appears that IgE sensitization to albumins occurs most frequently by the respiratory route. It has been reported that IgE recognition of Fel d 2 is more frequent in patients with atopic dermatitis (10). While this could indicate that it may be possible that sensitization can occur by the cutaneous route, it may equally be a mere sign/biomarker of strong IgE sensitization to albumin in such patients and/or broad IgE sensitization to animal allergens in general, including also albumins. Such allergens have been described also for other allergen sources as possible biomarkers for severe forms of allergy (56–58). However, IgE recognition of these biomarker allergens does not always seem to contribute to symptoms, rather it seems that broad IgE sensitization including these biomarker allergens is associated with the co-occurrence of several different allergic symptoms and severity of disease (59).

Albumin appears to have little resistance to gastrointestinal digestion and/or heating, which would be features of class 1 food allergens to which patients become sensitized by the oral route (60–62). Nevertheless, primary oral sensitization to albumins may occur, particularly when albumin is part of allergen sources that are rich in other proteins which may protect albumin against digestion. A typical example is sensitization to cow´s milk which contains besides albumin many other proteins and allergens that are highly resistant to digestion (63). In order to answer the question of whether oral sensitization to albumin present in food (e.g., milk, meat) has occurred via the gastrointestinal route molecular IgE diagnosis can be helpful. Thus certain patients may become sensitized via the gastrointestinal tract, whereas others become sensitized by the respiratory tract and it seems that the levels of allergen-specific IgE, their avidities, and the extent of cross-reactivity determine the evolvement, quality, and extent of clinical symptoms to albumins from different sources. We argue that it is useful to determine IgE levels and study IgE cross-reactivity to different albumins, for example by IgE inhibition experiments to determine the genuine allergen source which is responsible for albumin sensitization.

Such a diagnostic procedure may help clarify what type of IgE sensitization dominates in the various clinical syndromes that are attributed to IgE cross-reactivity to albumins (**Table 3**) (9, 12, 28, 31–34, 38, 39, 44, 64–87). These syndromes include for example the cat-pork/pork cat syndrome with gastrointestinal symptoms or skin symptoms in cat allergic patients when they eat pork or prepare pork meat dishes (28, 31, 39, 64–67). IgE sensitization to Fel d 2 may result in IgE cross-reactivity to BSA and mistakenly lead to the incorrect diagnosis of cow´s milk allergy. Simultaneous IgE reactivity to albumins from animals may be due to IgE cross-sensitization due to primary IgE sensitization to one respiratory allergen source associated with subsequent IgE cross-reactivity with albumins from other allergen sources or it can be a result of co-sensitization. Accordingly, molecular diagnosis with albumins from different allergen sources may provide information about the genuinely sensitizing allergen source and guide the prescription of allergen-specific immunotherapy (AIT). In the case of primary IgE sensitization to albumin from one allergen source AIT only to the genuinely-sensitizing source is indicated whereas in the case of co-sensitization AIT with the co-sensitizing allergen sources may be indicated if clinical relevance of the IgE sensitization has been confirmed. In this case, detection of genuine sensitization may be also confirmed by determining IgE sensitization against the genuine marker allergens of the different allergen sources (e.g., Fel d 1 for cat allergy) but this may not always be clear because even major cat and dog allergens may cross-react as shown for Fel d 7 and Can f 1 (88).

**Table 3:** Clinical syndromes due to albumin cross-reactivity.

Cross-reactive albumins	Syndrome or association	References
**Fel d 2 and Sus s 1**	Cat-pork syndrome/Pork-cat syndrome	(28, 31, 39, 64–67)
**Fel d 2 and Bos d 6**	Cat-milk	(32–34, 68–74)
**Fel d 2 and Can f 3**	Cat-dog	(9, 12, 75–78)
**Bos d 6 and Fel d 2**	Allergy in artificial insemination	(79–81)
**Sus s 1 and Gal d 5**	Pork-chicken	(64)
**Equ c 3**	Horse milk/mare dander	(82)
**Gal d 5**	Bird-egg/Egg feather	(44, 64, 83, 84)
**Bos d 6**	Cow milk/cow dander/beef	(70, 72, 73, 85)
**Equ c 3 and hamster albumin**	Horse meat allergy associated with allergy to hamsters	(38)
**Fel d 2 and Equ c 3**	Horse meat/cat dander	(86)
**Equ c 3 and Can f 3**	Horse meat/dog dander	(87)

**Table 3** provides a summary of clinically described syndromes in which IgE cross-reactivity to albumin plays a role (9, 12, 28, 31–34, 38, 39, 44, 64–87). The symptoms resulting from IgE cross-reactivity to albumin include the cat-pork syndrome, the cat-milk syndrome, cat-dog sensitization, allergy during artificial insemination with BSA-containing materials, the pork-chicken syndrome, the horse milk-dander syndrome, the bird-egg/egg-feather syndrome, the cow´s milk-dander-beef syndrome and horse meat allergy associated with allergy to hamsters, cats, and dogs (9, 12, 28, 31–34, 38, 39, 44, 64–87). Due to the large amounts of serum albumin in the circulatory system, connective tissues, feathers or hair, and egg yolks of birds, highly sensitive individuals develop symptoms after exposure to feathers or hair, shed by pets, and, subsequently develop symptoms when the patient is re-exposed to foods containing albumin. These symptoms are usually gastrointestinal in nature but sometimes include the skin or respiratory system as well.

The cat-pork syndrome has been widely reported in recent years, usually in people with respiratory allergies to cat dander or hair who develop acute allergic reactions to pork. It was first reported for patients eating pork after being exposed to cat hair and shown to cause digestive or even systemic allergies (28, 39, 65–67). Although cat serum albumin is not considered to be the major allergen triggering cat allergy (only 14%-23% of cat allergic patients are sensitized to Fel d 2), it is considered to be an important diagnostic marker considering that about one-third of cat allergy patients may experience symptoms upon pork ingestion (28). Cases of occupational exposure resulting in the development of cat-pork syndrome in meat processing employees have also been reported (64).

It is also possible that cat-allergic patients contain anti-BSA IgE antibodies reacting with milk. J Vicente-Serrano (85) and Karsonova (50) previously reported that patients allergic to BSA in milk may develop cross-reactivity to mammalian meat or epithelial cells, and that serum from patients allergic to cats reacts with BSA in milk.

Likewise, it was reported that cat-allergic patients had a severe anaphylactic reaction to BSA after artificial insemination with BSA-containing sperms, and after measurement of serum, IgE reactivity to serum albumin from other mammals such as cats, dogs, and pigs was found (81). Similarly, patients allergic to cats were found to have systemic symptoms due to an allergy to BSA in artificial insemination (80).

The bird-egg syndrome was first reported in 1985 when a parrot owner developed respiratory and cutaneous allergic reactions after ingesting egg yolk (83). After inhibition tests conducted by IgE-immunoblotting, serum albumin (Gal d 5), which is present in both egg yolk and parrot feathers, was identified as the relevant allergen. In fact, some patients show gradual remission of allergy to bird serum albumin after cessation of contact with bird feathers (83, 84).

For all the aforementioned syndromes, measurement of IgE levels specific for a panel of albumins from the different allergen sources by molecular diagnosis and eventually IgE inhibition experiments will in addition to the measurement of IgE sensitization to source-specific marker allergens provide information about the genuinely sensitizing allergen source for precision-medicine-based approaches for treatment and prevention.

## Allergy to albumin may also occur in animals

Several observations suggest that animals may develop immunological hypersensitivity reactions to HSA. BSA and HSA have been shown to be highly immunogenic in mice in fundamental studies in immunology (89–91). Furthermore, it has been reported that dogs and/or cats receiving HSA-containing solutions developed Type III hypersensitivity reactions and formed HSA-containing immune complexes (92–95). Likewise, it was found that chronic glomerulonephritis (GN) could be induced in N/M mice by daily injections of HSA. In these mice, the glomerular lesions were similar to that observed in human membranous GN and could be characterized by intense mesangial and capillary loop immunofluorescent staining for HSA, IgG, and C3 (96).

Subcutaneous sensitization with Aluminum-hydroxide-adsorbed Can f 3 was found to induce strong Can f 3-specific IgE responses as well as Can f 3-specific IgG responses in a murine model of IgE-mediated allergy (11). HSA has been also used to sensitize rats to demonstrate that IgE sensitization can be prevented by feeding HSA via oral tolerance induction (97). Similar results were obtained by feeding BSA which prevented the development of BSA-specific hypersensitivity (98).

The aforementioned examples thus demonstrate that it is possible to induce immunological hypersensitivity and in particular IgE-mediated hypersensitivity against albumin in experimental animal models. Moreover, certain of these animal studies demonstrate that it is possible to prevent the induction of albumin-specific hypersensitivity by the induction of oral tolerance (i.e., pre-feeding of animals with albumin before sensitization).

## Allergy to albumin: a case of failed tolerance or of highly selective tolerance?

**Figure 4:**
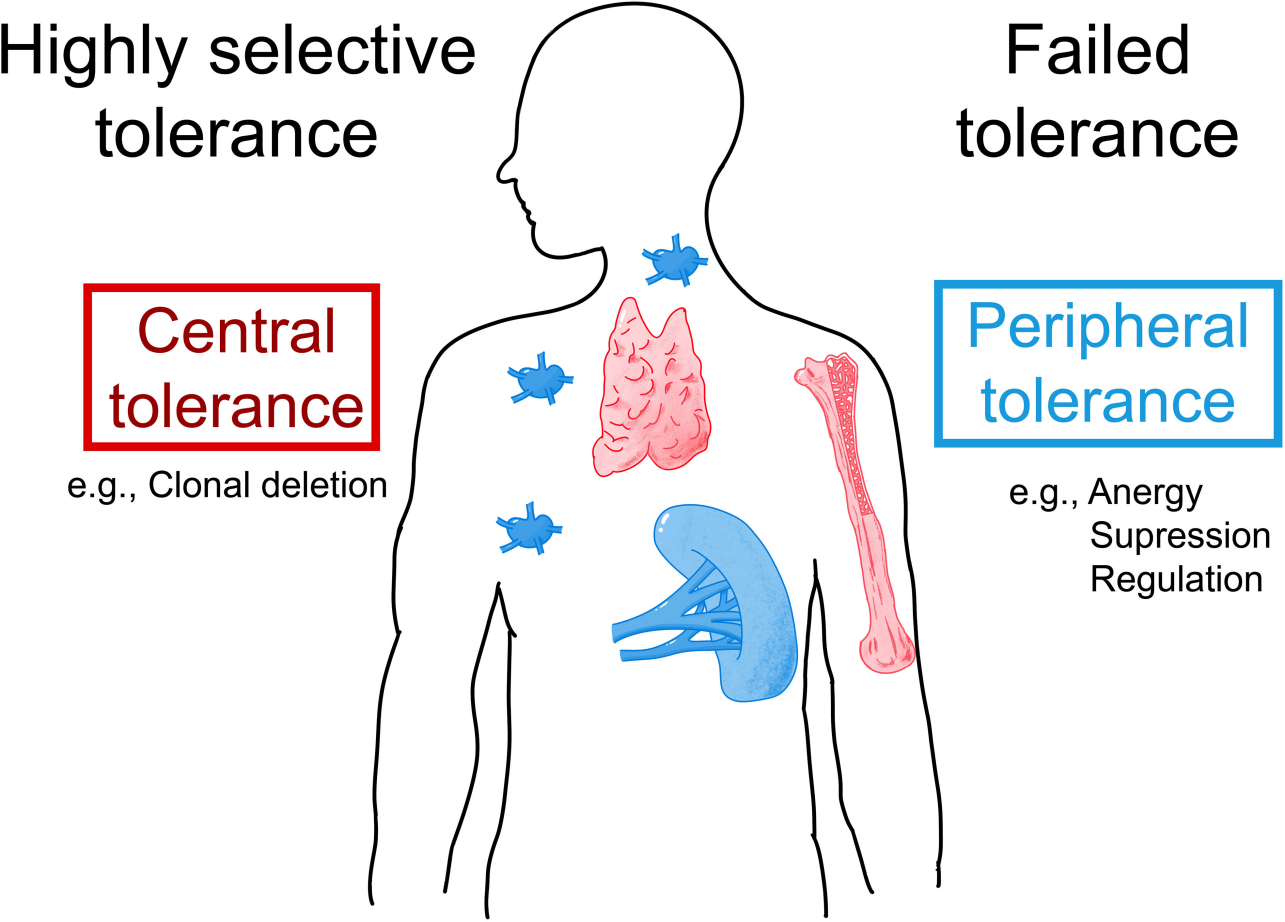
Possible mechanisms underlying IgE sensitization to albumins. Allergic patients who are allergic to exogenous albumins such as Fel d 2 and Can f 3 may lack IgE auto-sensitization to HSA due to highly selective central and/or peripheral tolerance to HSA (left part). Alternatively, patients with an allergy to exogenous albumins may show IgE- and T-cell cross-reactivity to HSA due to failed tolerance (right part). AIT with albumin-containing vaccines may induce and/or enhance auto-reactivity to HSA.

Due to the similarity of HSA and animal albumins, it is quite possible that patients allergic to animal albumins also contain antibodies and T cells reactive to HSA. Indeed, the appearance of anti-HSA antibodies in patients with systemic lupus erythematosus has been reported (99). Furthermore, the presence of IgM-HSA immune complexes has been described in patients with liver disease and autonomic dysfunction (100, 101). Yet another example of possible HSA-mediated auto-reactivity is the occurrence of anti-HSA autoantibodies in patients with autoimmune bullous skin diseases (102). The development of auto-reactivity and thus the failure of HSA-specific tolerance (**Figure 4**) may be explained by at least two mechanisms. First, it is possible that auto-reactivity is induced by an altered self. For example, it has been shown that one can induce anti-albumin autoantibodies by immunization of animals with self-albumin which has been altered by chemical means (103, 104). In agreement with these animal models, autoantibodies specific for chemically modified HSA have been described in patients suffering from autoimmune diseases (105–107). The second possibility for the induction of adaptive immune responses against HSA patients is cross-reactivity between a structurally related foreign antigen such as Fel d 2, Can f 3, Bos d 6, or other exogenous sensitizing animal albumins. In this context, it should be noted that cases of diabetes and nephropathy due to cross-reactivity of BSA and antigenic determinants on human cells have been reported (108–112).

IgE-auto-reactivity due to cross-reactivity of foreign antigens (113–117) as well as IgE-auto-reactivity to intracellular human antigens which do not share known structural similarity with exogenous antigens (118–120) have been previously described in allergic patients. Hence, IgE auto-reactivity is known to occur in allergic patients (121).

However, so far the presence of IgE antibodies specific to HSA could not be demonstrated and we are not aware of attempts to search for HSA-specific T cells in albumin-allergic patients. The question is therefore what the reason could be that so far no IgE antibodies specific for HSA could be detected. If there were HSA-specific IgE antibodies in albumin-allergic patients, these antibodies would occur in extremely low amounts. Given the high abundance of HSA in the blood HSA-specific IgE antibodies would therefore be completely adsorbed by HSA in the form of immune complexes and accordingly will not react in tests measuring IgE reactivity to solid phase bound HSA. The only possibility to detect HSA-reactive IgE would then be to try isolating such putative IgE-HSA immune complexes but this has not yet been done successfully. If indeed such IgE-HSA immune complexes and HSA-specific T cell responses could be found in albumin-allergic patients one may consider failed immunological tolerance as a reason for albumin-specific IgE and T cell responses in albumin–allergic patients (**Figure 4**). If failed tolerance can be demonstrated, it will be interesting to investigate if auto-reactivity to HSA may contribute to inflammation in albumin-allergic patients.

The second possibility is that patients allergic to exogenous albumins are selectively sensitized to the exogenous albumins, but not to HSA due to highly selective tolerance (**Figure 4**). In this case, albumin-specific T cells and IgE would selectively react with epitopes that differ between HSA and the exogenous albumins. Accordingly, mapping of IgE and T cell epitopes of exogenous albumins would need to be done in allergic patients sensitized to these albumins to clarify whether sensitization is due to highly selective central and/or peripheral tolerance which the latter may also include the activity of B regulatory cells (122–124). In this context, it will be very interesting to determine how much the extent of the putative selective tolerance depends on differences in sequence and structure between homologous antigens. This will be important for the development of therapeutic strategies for the treatment and prevention of allergy based on tolerance induction and in particular for the selection of tolerogenic peptides for such approaches (125–127).

## Can allergen-specific immunotherapy with albumin-containing vaccines induce auto-reactivity or even autoimmunity?

Besides the induction of T cell-based tolerance, AIT based on vaccines containing allergens or recombinant allergen derivatives is another possibility for allergen-specific treatment and prevention of allergy (128). The induction of allergen-specific IgG-blocking antibodies seems to be an important underlying mechanism of AIT (129). Vaccine-induced IgG antibodies are supposed to block allergens to induce IgE-mediated effector cell (e.g., mast cells, basophils, T cells) activation and boost IgE secondary IgE production (128). Currently, allergen extracts derived from natural allergen sources are mainly used for treatment (130). In the case of allergy to animal dander, especially for cat and dog allergy, natural allergen extracts that may contain albumin are used. It is thus possible that vaccination with albumin-containing allergen extracts may induce antibody and T cell responses not only against the corresponding exogenous albumins present in the vaccine but eventually also break tolerance resulting in the induction of HSA-specific antibody and T cell responses which then may lead to auto-reactivity in vaccinated subjects. At present there is no evidence for such a possibility but this topic has not yet been investigated intensively. In fact, to the best of our knowledge, it has not been studied systematically if special complications have been reported for albumin-sensitized allergic patients who had undergone AIT with albumin-containing allergen extracts. However, it has been shown that there are considerable side effects in patients who had received cat-specific AIT but it will be interesting to study if side effects due to auto-sensitization are more in albumin-reactive patients (131). Using modern molecular allergy vaccines based on recombinant allergens, recombinant allergen derivatives or allergen-derived peptides (128) will clearly be possible to eliminate the risk of inducing adaptive immunity to HSA by not including albumin and albumin-derived epitopes in the vaccines. However, it may be important to develop safe vaccines for patients with clinically relevant sensitizations to albumin. Accordingly, understanding the mechanisms of IgE-mediated allergy to albumin will be a prerequisite for the development of adequate and safe treatment strategies.

## Conclusions and open questions

Albumins from various animals, especially from cats and dogs, are important allergens for allergic patients with respiratory allergy and may contribute to symptoms of food allergy upon intake of albumin-containing food. Despite a high degree of sequence and structural similarity among exogenous albumins and human serum albumin, it is not known whether albumin-allergic patients show HSA-specific auto-reactivity or have developed selective tolerance. Unraveling the mechanisms and epitopes involved in allergy to albumins is important for at least two reasons. First of all, molecular diagnostic tests based on albumins from different sources may help to identify the genuinely sensitizing allergen source and guide precision medicine-based forms of allergen-specific forms of treatment and prevention. Second, knowledge regarding possible auto-reactivity to albumin in sensitized albumin allergic patients and about the potential risks of inducing albumin-specific auto-reactivity may help improve allergen-specific forms of treatment and prevention of patients allergic to animals.

## Author contributions

ZL and DT contributed to the design of the study, interpreted the findings, and wrote the manuscript. RV, AlK, and MK designed the study and contributed to the interpretation of the findings, writing and revising the manuscript. ZL, DT, IT, KR, AnK, EK, OE, MK, T-HC, MF-T, AlK, and RV provided materials, references, and/or figures/Tables, and read and revised the manuscript. All authors contributed to the article and approved the submitted version.
